# Construction Notes

**Published:** 1904-12-10

**Authors:** 


### CONSTRUCTION NOTES.

A new hospital has been opened at Crewkerne. The accommodation is for 15 patients, and the cost about £5,300.

The Westminster Hospital and the North-West London have benefited through the will of the late Captain W. O. Bird of Brighton.

Mr. R. Ogilvy has presented £1,000, on behalf of himself and his brother and sister, to the Royal Victoria Hospital, Dundee. The money will be devoted to the scheme to provide a cancer ward at the hospital.

A new infirmary has been opened in connection with the Aston Union Workhouse, near Birmingham. Part of the infirmary is old school buildings converted. The accommodation is for 124 patients at a cost of £6,000.

The foundation-stone has been laid of a new infirmary at the Exeter City Workhouse. It consists of five blocks and administration building. The accommodation, which includes 13 beds for consumptives, is for 152 patients. The cost is estimated at £10,500.

A new maternity hospital has been opened in Belfast. The charity was founded in 1793, and has moved and enlarged the premises until it has been found necessary to erect the present buildings, which contain four wards of four beds and several smaller wards for one and two beds.

